# Comparison of Hospital Consultation and Summer Camp Lifestyle Intervention Programs for Sustained Body Weight Loss in Overweight/Obese Greek Children

**DOI:** 10.3390/children9010086

**Published:** 2022-01-08

**Authors:** Anna L. Papageorgiou, Vasiliki Efthymiou, Aikaterini Giannouli, Paraskevi Xekouki, Christina C. Kranioti, George P. Chrousos

**Affiliations:** 1 University Research Institute of Maternal and Child Health and Precision Medicine, National and Kapodistrian University of Athens, 11527 Athens, Greece; anna_diet@yahoo.gr (A.L.P.); chrousge@med.uoa.gr (G.P.C.); 2 Center for Adolescent Medicine and UNESCO Chair on Adolescent Health Care, First Department of Pediatrics, School of Medicine, National and Kapodistrian University of Athens, Aghia Sophia Children’s Hospital, 11527 Athens, Greece; giannouli.katerina@gmail.com; 3 Department of Endocrinology, School of Medicine, University of Crete, 70013 Heraklion, Greece; p.xekouki@gmail.com; 4 Postgraduate Course on the Science of Stress and Health Promotion, School of Medicine, National and Kapodistrian University of Athens, 15772 Athens, Greece; chr1s_kran@hotmail.com

**Keywords:** obesity, camp, nutrition, physical activity

## Abstract

Two lifestyle intervention programs of a health initiative named “Evrostia” were conducted at (a) an outpatient obesity clinic of a children’s hospital and (b) summer camp (SC), respectively. Thirty overweight/obese children were randomly selected to participate in each intervention arm to assess the efficacy of the SC intervention and its possible superiority over usual hospital consultation (HC) practice. There was a statistically significant decrease in body weight (BW), and body mass index (BMI) in both programs. A higher duration of reduced BW was observed in the SC compared to HC intervention. Regarding the nutritional behavior, there was a significant increase in the consumption of breakfast, fruit and vegetables, and a reduction in the consumption of beverages and sweets in the SC group. A significant increase in the hours of weekly physical activity was also observed in children of the SC program. The comparison between the two lifestyle intervention programs showed that the SC program improved nutritional behaviors and physical activity and promoted longer preservation of BW loss than that of the HC program. Thus, the holistic and experiential approach of the SC program was more successful in the treatment of overweight and obesity in children than a conventional HC program.

## 1. Introduction

The World Health Organization (WHO) characterizes obesity as a nutritional disorder, which has developed into an epidemic, affecting both children and adolescents and threatening public health with its physical and mental implications [1]. Body mass index (BMI) is used to assess excess body fat and set the cut-offs that define obesity [2]. Worldwide, one in five children and adolescents is obese [3]. In the context of the Childhood Obesity Surveillance Initiative of the WHO, a Greek prevalence study found that 25.8% of seven-year-old boys and 19.7% of seven-year-old girls are obese by the WHO definition [4]. In 2019, Spinelli published a report of obesity prevalence in 21 European countries, including Greece. Greece was among the countries with the highest prevalence of severe obesity (BMI-for-age above +3 Z-scores relative to the 2007 WHO growth reference median). Total severe obesity prevalence in Greece was reported 4.8% (95% CI 1.4–5.7), much higher in boys 7.2% (95% CI 6.2–8.5) than in girls 2.4% (95% CI 1.6–3.3) [5]. Systematic analysis data seem more ominous, raising overweight/obese prevalence to 33.7 (29.6–37.7) for boys and 29.1 (25.3–33.1) for girls [6].

Obesity is a multifactorial disorder, as its development and progress are influenced by genetic, epigenetic, and environmental factors. Influencing factors include the body weight of the parents, especially of the mother, the birth weight, excessive nutrition, as well as under-nutrition of the fetus at critical periods, gestational diabetes associated with baby macrosomia. Moreover, mother’s nutrition during pregnancy, as well as breastfeeding vs. bottle feeding, with the former acting protectively against the development of obesity [7,8,9]. Dietary, environmental, and behavioral factors increase the childhood obesity epidemic [10,11]. The nutritional habits of Greek children 3–18 years old, are characterized by low adherence to a Mediterranean diet and increased consumption of free sugars in sweets, non-homemade bakery products, and sugared drinks, and consumption of fast-food regularly [12,13,14]. In relation to physical exercise, almost half of Greek children stand below the recommended duration of at least one hour per day of physical activity [13,14]. Additionally, excessive screen time and having meals in front of a screen have been associated with obesity lifestyle patterns [12]. Short sleep duration, going to bed late, and skipping breakfast, are also contributing factors [14,15].

Various approaches have been utilized to help overweight and obese children adopt healthier lifestyles. Most children are treated by a primary care physician, while more severe cases require a multidisciplinary approach by a physician, a dietitian, and a psychologist [2,16]. The usual approach starts with a careful evaluation of current problematic dietary and fitness habits. Goal setting follows, regarding both nutrition (structured meal plan, portion sizes, consuming fruit and vegetables, and limiting energy-dense foods) and physical activity. Weight goals are not usually used in children. Behavioral strategies are used in order to change current patterns and promote lifestyle modification for the whole family [2,17]. Summer camps provide a structured, safe, and supportive environment for a holistic lifestyle intervention. Most of these programs combine a dieting, exercising, and behavioral modification approach. Summer camp interventions have proved their efficacy in many studies not only in weight loss and physical activity but also in other metabolic parameters such as systolic and diastolic blood pressure [18,19,20]. Health-related quality of life was also improved [21].

We conceptualized our intervention to assess the efficacy of immersive summer-camp interventions in parallel to the conventional hospital consultation programs that are usually applied in Greece. Given the extra cost of sleepaways and camps in general, efficacy and any additional advantages of such interventions should be carefully studied. The main scope of the study was to evaluate if a structured summer camp intervention is more efficient and provides a more durable effect than conventional outpatient consultation.

## 2. Materials and Methods

### 2.1. Procedure

The study is a prospective, randomized, interventional study investigating and comparing the effectiveness of educational-lifestyle intervention programs, conducted in different environments, an outpatient obesity clinic in a public children’s hospital vs. a summer camp. The study addresses the reduction in body weight, the modification of nutritional behavior, and physical activity of overweight/obese Greek children. The study took place in Greece, both at Aghia Sophia Children’s Hospital (January–July 2016), and at the sleepaway camp Evrostia at the Prefecture of Corinthia (June–July 2017). The study was approved by the ethics committee of the hospital.

### 2.2. Participants

**Figure 1:**
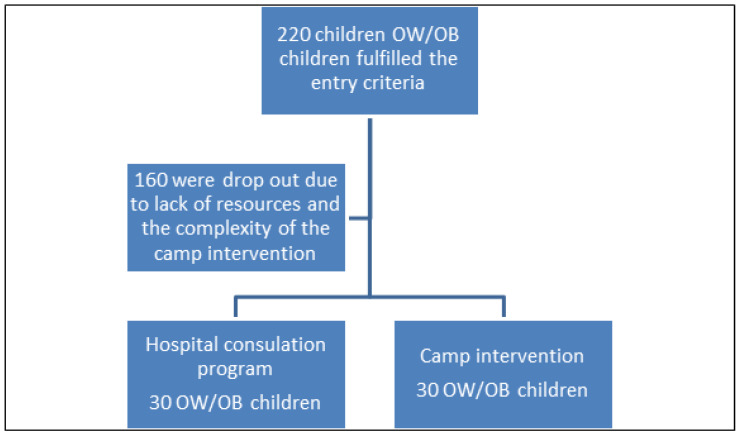
Flowchart of children recruitment for both intervention programs. OB; obese, OW; overweight.

A total of 220 children aged 7–12 years old with increased body weight (>85th percentage place of the Greek growth diagrams), habitants of Attica, who visited the Pediatric Obesity Center of the first Dept. of Pediatrics of the National and Kapodistrian University of Athens and fulfilled the entry criteria mentioned below were invited to participate in the study (Figure 1). Out of 220 children, only 60 were accepted in the present study due to a lack of resources and the complexity of the camp intervention.

The entry criteria for the participants were: sufficient knowledge of the Greek language (fluent use of oral and written language), age between 7–12 years with excess body weight, >85th percentage at the Greek growth diagrams, absence of metabolic diseases or genetic syndromes, absence of a psychiatric disorder history, and no psychoactive substance use. Patients and their parents were informed about the study and a consent form was signed. Participants were given no inducements for their participation in the research study, while their parents were fully informed of the aims and procedures of the protocol. In addition, both parents and children received affirmation regarding confidentiality issues, as well as their right to stop their participation in the research at any time, without having to report the reasons for their decision.

For the camp lifestyle intervention, 30 children from the cohort were selected randomly and invited to participate in a 3-week summer camp program of nutritional training and physical exercise the following year. The other 30 children underwent the hospital consultation intervention.

### 2.3. Measures

Anthropometric and nutritional evaluation was conducted in all children, while the nutritional habits and the weekly duration of physical activity were documented. Sociodemographic data, as well as data related to the medical history of the children, were collected through a questionnaire. The anthropometric evaluation included measurement of weight, height, and calculated body mass index (BMI). Body weight was measured without shoes, dressed only with underpants, using a Sega scale with accuracy +/−200 g. Height was measured at the standing position, also without shoes, using the Sega height scale incorporated in the weight scale. Body mass index (BMI) was calculated as kilograms per square meter (kg/m^2^), according to the formula BMI = Weight/(Height)^2^. The categorization of the children was done according to the Greek Growth Diagrams.

The nutritional evaluation was conducted through the Food Frequency Questionnaire (FFQ) and the 24 h Recall (of the Food Documentation Diary). The version of Dr. Gladys Block’s Food Frequency Questionnaire (FFQ) was employed [22]. Moreover, a 24 h recall obtained from the United States Department of Agriculture (USDA) automated multiple-pass method was used [23]. For fitness evaluation, we employed the seven-fay physical activity recall (PAR) [24].

### 2.4. Hospital Consultation Program

The monthly nutritional plan was designed based on the principles of a balanced diet that a child must adhere to according to his/her age, sex, and daily activity. Specifically, the recommendations that were followed were 55–60% carbohydrates, 15–20% protein, and 25–30% fat. The diet plan was not strictly caloric restricted. High glycemic index foods, such as sweets, soft drinks, and natural juices, were excluded. In addition, a restriction on the consumption of saturated fat was proposed mainly through baby foods (biscuits, croissants, ice creams). Children were encouraged to consume fiber-rich foods, such as fruits, vegetables, and whole grains. Written instructions on food choices and cooking were provided, including easy ways to prepare a healthy breakfast and other meals. Finally, physical activity recommendations and examples of how a child could achieve 60 min of motion a day were provided.

### 2.5. Camp Intervention

The design of the camp health promotion intervention included a combination of experiential methods and active participation with the help of a child psychologist and a dietician. More specifically, the program implemented active participation of children in the preparation of the meals with the help of a specialized chef in the kitchen. The children made their own breakfast choices and obtained information on food groups and nutrients at the same time. The children were then divided into groups, each with the help of a chef to prepare the meals of the day. The children selected the combinations and meals, and their choices were evaluated and commented on by other children’s groups. The goal was to select low glycemic index foods high in fiber, less salt and sugar, and to know which foods are high in cholesterol, saturated fats, and trans fats.

The basic guidelines for food selection were given in writing after an educational program using audiovisual media. At the same time, the program included an art workshop, where children painted and prepared a play with the theme “Health-Nutrition-Exercise”. With the help of a psychologist, knowledge games were designed to give children more information about food and the value of exercise for health, i.e., card games with each card providing information on healthy food replacements (for instance, frozen yogurt instead of ice cream). At pre-scheduled hours of the day, audiovisual materials, including videos, were shown addressing the basic functions of the human body, such as digestion and metabolism. As for the physical activity part of the camp intervention, we aimed to stress the importance of exercise in health and body weight management and to convey the message that a minimum of 60 min per day of moderate to vigorous physical activity should be part of a healthy lifestyle [25,26]. The camp program included the following: (i) Fitness program: 2 h of morning workout and 2 h of afternoon work; this included 30 min of aerobic exercise, 30 min of strength training and 1 h of free play; (ii) athletic activities program: ground and in-water exercises, such as walking, swimming, aqua aerobic, cycling, jogging, climbing and horse riding; (iii) participatory sports and leisure activities, such as basketball, soccer, or volleyball and free improvised play.

At the end of the intervention, the children received their own personal file with records of all the activities they participated in, as well as useful practical tips to avoid mistakes in their daily diet. Their parents were informed of the children’s progress and drawbacks, and recommendations for home nutrition were provided. On the last day of the hospital consultation program and camp intervention, all measures were repeated. After that, repeated measurements took place at 1, 2, 4, and 6 months later with the purpose to evaluate the maintenance of the result.

### 2.6. Data Analyses

Statistical analyses were conducted using the SPSS software 22 version for Windows (IBM Corp. Released 2013. IBM SPSS Statistics for Windows, Version 22.0. Armonk, NY, USA: IBM Corp.). *p*-values were based on two-sided tests and statistical significance was set at *p* < 0.05. Continuous variables are shown as the mean ± standard deviation, whereas absolute frequencies or percentages were calculated for discrete variables. Normality was tested by using the Kolmogorov–Smirnov test and normal probability plots. Differences between patients and controls were calculated using Student *t*-tests after assessing the homogeneity of variance. Discrete variables were compared with the chi-square test. Comparisons between continuous or discrete data at baseline and after the intervention were carried out with the use of a paired *t*-test and a McNemar’s test, respectively.

## 3. Results

Thirty children participated in the camp intervention: 14 boys (47%) and 16 girls (53%). Participants in camp and hospital consultation intervention were chosen to be sex- BMI- and age-matched. The mean age ± SD was 10.9 ± 2.2 for the total sample. Participants’ anthropometric characteristics are shown in Table 1. Children in the hospital and camp intervention programs had matched BMI percentiles; 13.3% were between the 70–90th BMI percentile, 43.3% in the 90–97th BMI percentile, and the rest 43.3% were in the exceeded 97th BMI percentile.

Statistically significant variations were observed before and after the interventions in the camp and hospital (Table 2). Body weight, BMI, BMI z-score decreased after both interventions (*p* < 0.001 for all). BMI and BMI z-score also had a significant absolute reduction in both hospital and camp interventions (*p* < 0.001 for all). A greater absolute change in body weight was observed (*p* < 0.001) in the camp (−2.13 ± 1.22 kg) than in the hospital consultation program group (−0.83 ± 0.83 kg). Apart from the absolute change in BMI and body weight of the children that participated in the two interventions, we examined the time interval that children managed to keep their body weight down. The children who participated in the hospital consultation program achieved to keep their body weight down for 2.0 ± 1.8 months, whereas the participants in the camp program kept their weight down for 4.2 ± 1.4 months, which was significantly longer.

Regarding the diet and lifestyle habits of the participants (Table 2), the study showed a greater positive change in physical activity in children of the camp program (1.33 ± 0.88) than that those of the hospital program (0.00 ± 0.00; *p* < 0.001). The absolute change in sweet and soda consumption in the camp program’s children was greater (−1.17 ± 1.23 and −2.57 ± 1.57, respectively) than those of the hospital program (−0.40 ± 0.97 and 0.00 ± 0.00, respectively with *p* = 0.007 and *p* < 0.001, respectively). Moreover, the percentage of participants who had daily breakfast, fruit and vegetable increased significantly following the camp intervention program (*p* < 0.001, *p* < 0.001, *p* = 0.031, respectively), but not in the hospital program. The opposite pattern was observed for weekly delivery meals from 60% to 30% (*p* = 0.004) after the camp intervention program.

## 4. Discussion

Participation in summer camps for obesity is a form of immersion therapy, which has been proved effective in the majority of studies [27]. They date more than 50 years back and they put participants in a controlled, educational, and therapeutic environment, keeping them away from obesogenic behaviors. Their hypothesized success can also be justified by the “structured days hypothesis” which states that absences of daily school routine make summer a vulnerable period for weight gain, so it should be targeted by interventions [28]. A meta-analysis of 22 studies published in 2010, showed a 191% greater reduction in percent-overweight posttreatment and 130% in follow-up compared to outpatient interventions, and a lower attrition rate [27]. More recent randomized controlled trials, such as the one published by Benestad et al. did not find a difference in BMI and BMI SD scores between summer camp and outpatient intervention at 2-year follow up [29]. However, Health-related quality of life specific for adiposity appeared more improved in the summer camp group [21].

On the other side, both interventions in our study produced more optimistic results, in agreement with some of the literature. The participation of children in both the hospital consultation and camp program “Evrostia” resulted in a significant reduction in their body weight already after two weeks, with a greater reduction in the children of the camp program. This replicated the findings of the study by Marschhoff et al. and more recent studies [19,30,31,32,33]. The weakness of the classical programs to control body weight with nutritional interventions at primary care is probably because the children who receive nutritional counseling, to apply in their usual environment, results in limited conformity. On the other hand, the participation of children in programs outside their home, which provide recreational, educational, and experiential stimuli, allows lifestyle training on nutrition, exercise, changes in behavior. Indeed, the recreational character of these experiential programs and the group approach created a pleasant atmosphere associated with stress reduction, allowing better learning.

Another study by Larsen et al. evaluated the effectiveness of a six-week multicomponent immersive day-camp and an ensuing family-based intervention, body weight-loss intervention for overweight and obese children. The authors demonstrated that in 6 weeks, children of the day-camp intervention had improved BMI and all secondary outcomes when compared to children that received standard intervention [20]. Significant pre–post waist-to-height reductions were also observed in a fitness camp with reductions maintained for 2 months, in contrast to children in the comparison group in whom an increase in the body weight was actually observed [34].

Significant changes were observed in the nutritional behavior and habits of children in our camp program. After three weeks in the camp, there was increased consumption of breakfast and intake of fruit and vegetables, and reduced consumption of sweets and beverages, similar to other studies [35,36,37,38,39]. The presence of social support and the creation of strong interpersonal networks favor interchange of information and experiences, and collaboration and participation in new activities, which contribute to the adoption of healthy behaviors by overweight and obese children [40]. In addition, getting away from the home environment, which might have contributed to their development of obesity, and finding themselves in a different supportive environment with children of the same age, allows them to proceed with a new shared goal through a conscious change in their behavior.

Children who participated in our camp program increased their physical activity, which explains the greater body weight loss and its preservation for a longer period of time. Benefits of camp interventions through an increase in exercise have also been reported in other studies, in which an increase in aerobic exercise, improvement in fitness, and self-esteem were observed [19,40,41].

One of the limitations of this study is the size of the sample, which is relatively small. In addition, the short duration of the camp program and the lack of long-term follow up beyond 6 months of intervention of the participants beyond the 6-month post-intervention period are potential drawbacks. Finally, the role of the parents was not specifically studied, granted that probably their participation might have affected the home nutritional behavior of their children. Participation of parents could increase the long-term success of such programs; naturally, parents should be trained in parallel to the children to be able to manage everyday routine lifestyle issues [42,43].

## 5. Conclusions

Our prospective, randomized, interventional study confirmed the results of other studies that summer camp body weight and lifestyle interventions are effective. In some areas of interest, such as the duration of body weight loss maintenance after the intervention, camp participants demonstrated longer time periods before weight attrition. Nutritional and fitness habits followed a similar improvement pattern. Thus, the structured, immersive environment of a camp provides additional benefits and helps overweight and obese children adopt a healthier lifestyle and maintain a reduced, healthier body weight.

## Figures and Tables

**Table 1:** Characteristics of participants in camp intervention program (Ν = 60).

	Total Sample (*n* = 60)	Boys (*n* = 26)	Girls (*n* = 34)	*p*-Value
Age (years)	10.93 ± 2.18	11.69 ± 1.85	10.35 ± 2.25	**0.017**
Weight (kg)	65.77 ± 17.24	68.54 ± 15.51	63.65 ± 18.39	0.280
Height (cm)	151.63 ± 13.10	156.85 ± 13.40	147.65 ± 11.54	**0.006**
BMI (kg/m^2^)	28.07 ± 4.19	27.44 ± 3.02	28.55 ± 4.90	0.287

BMI indicates Body Mass Index. Values are expressed as mean ± standard deviation (SD). *p*-value calculated using Student *t*-test. Bold indicates statistically significant differences.

**Table 2:** Comparison of anthropometric parameters as an absolute change in diet habits and physical activity between camp and hospital intervention groups.

	Camp Intervention			Hospital Intervention			
	Before Intervention	After Intervention	*p* *	Before Intervention	After Intervention	*p* *	*p* ^§^
Gender (Male, %)	13 (43.3%)			13 (43.3%)			>0.999 ^†^
Weight (kg) ^a^	65.77 ± 17.38	63.63 ± 16.70	**<0.001**	65.63 ± 17.21	64.80 ± 16.74	**<0.001**	-
BMI (kg/m^2^) ^a^	28.07 ± 4.23	27.17 ± 3.88	**<0.001**	28.07 ± 4.23	27.21 ± 3.68	**<0.001**	-
BMI z-score ^a^	2.13 ± 0.30	2.06 ± 0.30	**<0.001**	2.13 ± 0.30	2.10 ± 0.30	**<0.001**	-
Absolute change in weight (kg) ^a^	−2.13 ± 1.22			−0.83 ± 0.83			**<0.001**
Absolute change in BMI (kg/m^2^)	−0.91 ± 0.50			−0.38 ±0.36			**<0.001**
Absolute change in BMI z-score ^a^	−0.08 ± 0.05			−0.03 ± 0.03			**<0.001**
Time duration of weight retention (in months) ^a^	4.17 ± 1.42			2.03 ± 1.79			**<0.001**
Physical activity score ^a^	2.46 ± 0.79	3.82 ± 0.77	<0.001 *	2.50 ± 0.82	2.50 ± 0.82	>0.999 *	-
Sweet consumption (per week) ^a^	3.13 ± 0.86	1.97±0.93	<0.001 *	3.13 ± 0.86	2.73 ± 0.98	0.031 *	-
Soda consumption (per week) ^a^	3.30 ± 1.29	0.73 ± 0.69	<0.001 *	3.30 ± 1.29	3.30 ± 1.29	>0.999 *	-
Absolute change in weekly sweet consumption ^a^	−1.17 ± 1.23			−0.40 ± 0.97			**0.007 ^§^**
Absolute change in weekly soda consumption ^a^	−2.57 ± 1.57			0.00 ± 0.00			**<0.001 ^§^**
Absolute change in physical activity score ^a^	1.33 ± 0.88			0.00 ± 0.00			**<0.001 ^§^**
Breakfast (yes, per day) ^b^	43.3	96.7	**<0.001 ^‡^**	60.0	73.3	0.125 ^‡^	
Fruit/day (at least one portion) ^b^	40.0	93.3	**<0.001 ^‡^**	40.0	56.7	0.063 ^‡^	
Vegetables/day (at least one portion) ^b^	76.7	96.7	**0.031 ^‡^**	76.7	76.7	>0.999 ^‡^	
Delivery (yes, per week) ^b^	60.0	30.0	**0.004 ^‡^**	80.0	80.0	>0.999 ^‡^	

BMI—body mass index. Values are expressed as ^a^ mean ± standard deviation (SD) or ^b^ frequency (%). *p*-value calculated using * paired *t*-test, ^§^ *t*-test, ^‡^ McNemar test, and ^†^ chi-square test. Bold indicates statistically significant differences.

## Data Availability

The datasets used in this study are available from the corresponding author.
